# Different Angiogenic Abilities of Self-Setting Calcium Phosphate Cement Scaffolds Consisting of Different Proportions of Fibrin Glue

**DOI:** 10.1155/2014/785146

**Published:** 2014-06-10

**Authors:** Jintao Xiu, Junjun Fan, Jie Li, Geng Cui, Wei Lei

**Affiliations:** ^1^Institute of Orthopaedics, Xijing Hospital, Fourth Military Medical University, No. 127 Changle West Street, Xi'an 710032, China; ^2^Institute of Gynaecology and Obstetrics, General Hospital of PLA, Beijing 100853, China; ^3^Institute of Orthopaedics, General Hospital of PLA, Beijing 100853, China

## Abstract

To investigate the different angiogenic abilities of the self-setting calcium phosphate cement (CPC) consisting of different proportions of fibrin glue (FG), the CPC powder and the FG solution were mixed at the powder/liquid (P/L) ratios of 1 : 0.5, 1 : 1, and 1 : 2 (g/mL), respectively, and pure CPC was used as a control. After being implanted into the lumbar dorsal fascia of the rabbit, the angiogenic process was evaluated by histological examination and CD31 immunohistochemistry to detect the new blood vessels. The result of the new blood vessel showed that the P/L ratio of 1 : 1 group indicated the largest quantity of new blood vessel at 4 weeks, 8 weeks, and 12 weeks after implantation, respectively. The histological evaluation also showed the best vascular morphology in the 1 : 1 group at 4 weeks, 8 weeks, and 12 weeks after the operation, respectively. Our study indicated that the CPC-FG composite scaffold at the P/L ratio of 1 : 1  (g/mL) stimulated angiopoiesis better than any other P/L ratios and has significant potential as the bioactive material for the treatment of bone defects.

## 1. Introduction

In the recent few decades, bone tissue engineering has developed very fast and gives us the hope to repair the large bone defect. But there are still many problems with the clinical application of tissue engineered bone. Bone tissue is a highly vascularized tissue and the bone cells need sufficient blood supply to maintain their nutrition [1]. Before the vascularization completed, the implanted bone graft gets nutrition mainly through the osmosis of interstitium fluid and only the bone graft surface can get nutrition [2]. The osteogenesis is influenced by the process of vascularization and this limits the clinical application of tissue engineered bone [3–6]. Therefore, to get a better reparative effect, the bone scaffolds should support angiogenesis and neovascularization to generate a capillary network with the capacity to deliver nutrients [7–9].

Calcium phosphate cement has been proved to be a suitable scaffold and has been widely used as bone tissue engineering scaffold to repair the bone defect [10, 11]. The self-setting calcium phosphate cement has also successfully been prepared with a good plasticity [12]. However, self-setting calcium phosphate cement also has the intrinsic disadvantages such as the brittleness and low strength which limits its use in the large weight-bearing bone defect [13]. More recently, coating fibrin glues into on the scaffold surface is an effective approach to improving the mechanical property of calcium phosphate cement [14, 15]. Fibrin glues are composed of fibrinogen and thrombin and widely used in surgery [16]. Some publications have reported that they can be used in the bone tissue regeneration with their osteoinductive and angiogenic properties [17, 18]. The combining of calcium phosphate cement and fibrin glues has been proven to enhance the proliferation and differentiation of mesenchymal stem cells and to induce bone tissue formation [19, 20]. And the composite scaffolds also have new biomechanical properties and can be used to repair the bone defects in in vivo studies [21, 22]. However, the angiogenic abilities of this composite scaffolds consisting of different proportions of fibrin glue have not been studied.

The aim of our study was to investigate the different angiogenic abilities of the self-setting calcium phosphate cement scaffolds consisting of different proportions of fibrin glue and to find the optimal proportion of the fibrin glue.

## 2. Materials and Methods

### 2.1. Preparation of Composite Scaffold

Preparation of the composite scaffold was done according to our previous studies [23, 24]. A commercially available SFDA-approved CPC powder (Shanghai Rebone Biomaterials Co., Shanghai, China) and FG (Bioseal Biotech Co, Guangzhou, China) were used. The CPC powder and FG solution were mixed at three different powder/liquid mass ratios (P/L) (g/mL): 1 : 0.5, 1 : 2, and 1 : 4 in sterile syringe. Each CPC-FG mixture was immediately injected into a cylindrical mold of 5 mm diameter and 10 mm length to make cylindrical scaffolds. Each specimen was set in a humidor with 100% relative humidity at 37°C for 4 h and then immersed in distilled water 37°C for 20 h. The pure CPC scaffold was made without the FG and also fabricated to serve as a control.

### 2.2. Animals and Surgical Procedure

32 adult male New Zealand White rabbits (provided by the Experimental Animal Center of the Fourth Military Medical University, Xi'an, China), weighing from 2.8 kg to 3.5 kg, were used in the study and randomly divided into four groups. The animal experiments were performed according to the protocols approved by the Institutional Animal Care Committee of the Fourth Military Medical University. General anesthesia was induced by intramuscular injection of 35 mg/kg ketamine. After shaving and disinfecting the dorsal area, the longitudinal incision of 6-7 cm length was performed in the dorsal skin of each animal. The subcutaneous tissues were dissociated to show the lumbodorsal fascia and the fascia was cut open deep into the muscular layer. And two composite scaffolds were implanted into the cavity and wrapped by the lumbodorsal fascia in each rabbit. After implantation, the wound was rinsed with saline and the incision was closed in layers. The rabbits were treated with penicillin (240,000 UI) for 3 days postoperatively. Two animals from each group were sacrificed using an overdose of sodium pentobarbital at 2, 4, 8, and 12 weeks after the operation. The material and surrounding muscular tissue of each animal were harvested and immerged in the neutral buffered formalin solution.

### 2.3. Histology

For the histology observation under the light microscopy, formalin-fixed specimens of the material scaffold and the surrounding muscular tissue were decalcified and embedded in paraffin. Then they were cut into 10 sections with 4 *μ*m thickness, and 5 sections of each specimen were stained with hematoxylin and eosin according to standard procedures.

### 2.4. Immunohistochemistry

The other 5 sections of each specimen were used for immunostaining of CD31. Incubation of the sections with 3% H_2_O_2_ for 10 min blocked the nonspecific binding. The antigen was repaired by heat. The sections were incubated with 10% normal goat serum in PBS for 20 minutes to increase the penetration of the antibodies. The sections were then incubated in the primary antibodies of anti-CD31 (1 : 100, Boster Wuhai Biological Technology Ltd., China) for 2 h and incubated in second antibodies for 20 min at room temperature. Then they were further processed using SABC immunostaining kit (Boster Wuhai Biological Technology Ltd) according to the manufacturer's instruction. Five images of each section were obtained randomly. Quantitative image analysis of CD31 was performed to determine the number of new blood vessels in the composite scaffold and the mean number of these new vessels stained by CD31 was calculated from all sections of each sample in each group. To limit counting bias, the total number of vessels was recorded with Image-Pro Plus software. A vascular section was defined as a vessel with a recognizable lumen. Any single endothelial cell or cluster of endothelial cells clearly separated from adjacent vessels was considered as one countable vessel. Areas of bleeding and fibrous were also disregarded. The evaluation was performed by three individuals blinded to the groups.

### 2.5. Statistical Analysis

All data were analyzed using SPSS software. The data were expressed as mean ± standard deviation (SD) and levels were compared by a one-way analysis of variance and Student's *t*-test. *P* values less than 0.05 were considered significant.

## 3. Results

### 3.1. Gross Observation

The incisions of the animals were well healed and there was no infection or death. At 2 weeks, the scaffolds were combined with the surrounding tissues loosely and could be stripped easily. At 4 weeks, the CPC-FGs scaffolds were combined with the surrounding tissues more tightly and there were blood vessels between the scaffold and the surrounding tissues. But the CPC scaffold was combined with the host tissue loosely. At 8 weeks, the surfaces of the CPC-FGs scaffolds became coarse, but CPC scaffolds did not change. At 12 weeks, all the scaffolds were compressed and the sizes were decreased.

### 3.2. Histological Assessment

According to the results of HE staining, there was no obvious inflammation in the host tissues and the implanted scaffold of all groups at different times.

**Figure 1:**
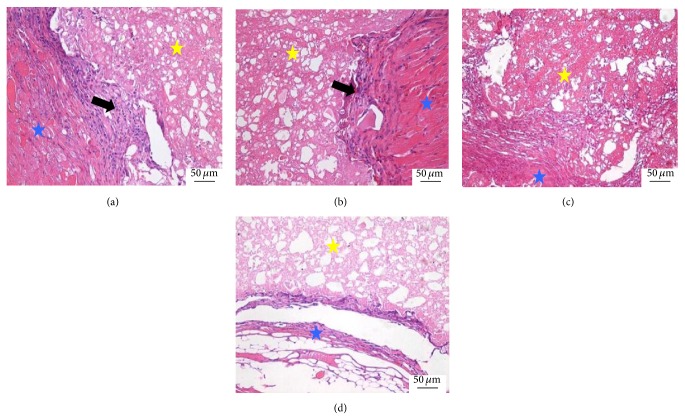
Histological morphologies after implantation at 2 weeks (HE^*^200). (a) The 1 : 1 P/L ratio of CPC-FG; (b) 1 : 0.5 P/L ratio of CPC-FG; (c) 1 : 2 P/L ratio of CPC-FG; (d) pure CPC. The yellow star was represented as the materials and the blue star was surrounding muscle tissue. The black arrow was the new blood vessel.

At 2 weeks, the scaffold was surrounded by the surrounding fibrous tissues and rare and naive blood capillary with a few erythrocytes in the blood lumen was shown in the interface between the scaffold and host tissues in all groups. The densities of the blood capillary in there CPC-FG groups were similar and all of them were higher than the pure CPC group (Figure 1).

**Figure 2:**
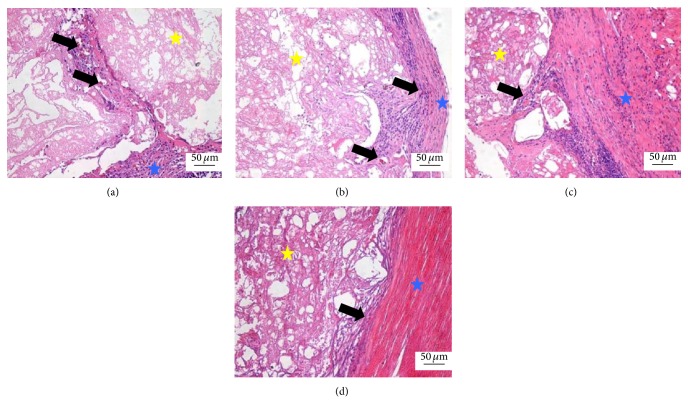
Histological morphologies after implantation at 4 weeks (HE^*^200). (a) The 1 : 1 P/L ratio of CPC-FG; (b) 1 : 0.5 P/L ratio of CPC-FG; (c) 1 : 2 P/L ratio of CPC-FG; (d) pure CPC. The yellow star was represented as the materials and the blue star was surrounding muscle tissue. The black arrow was the new blood vessel.

At 4 weeks, the scaffold was surrounded by more fibrous tissues and more blood capillary grew into the inside of scaffold. The blood capillary in the peripheral scaffold was more mature and the density was higher than in the internal scaffold in all groups. The densities of the blood capillary in these three CPC-FG groups were higher than the pure CPC group and were highest in the ratio 1 : 1 group (Figure 2).

**Figure 3:**
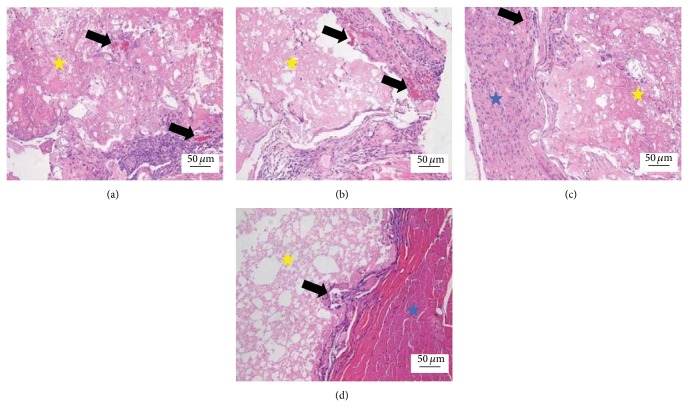
Histological morphologies after implantation at 8 weeks (HE^*^200). (a) The 1 : 1 P/L ratio of CPC-FG; (b) 1 : 0.5 P/L ratio of CPC-FG; (c) 1 : 2 P/L ratio of CPC-FG; (d) pure CPC. The yellow star was represented as the materials and the blue star was surrounding muscle tissue. The black arrow was the new blood vessel.

At 8 weeks, the fibrous tissues around the scaffold were increased, but the density of blood capillary was not obviously increased compared with 4 weeks. The structure of the blood capillary was more mature and the blood lumen was bigger than 4 weeks (Figure 3).

**Figure 4:**
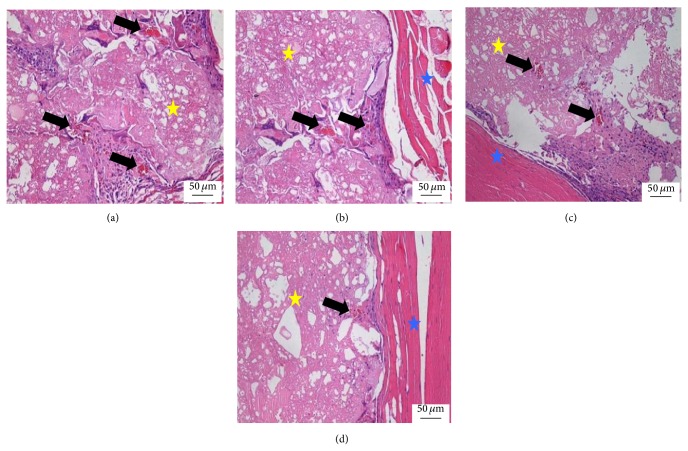
Histological morphologies after implantation at 12 weeks (HE^*^200). (a) The 1 : 1 P/L ratio of CPC-FG; (b) 1 : 0.5 P/L ratio of CPC-FG; (c) 1 : 2 P/L ratio of CPC-FG; (c) pure CPC. The yellow star was represented as the materials and the blue star was surrounding muscle tissue. The black arrow was the new blood vessel.

At 12 weeks, the scaffold was contracted and the size was decreased compared with 8 weeks. The surrounding fibrous tissues and the density of blood capillary were not obviously changed compared with 8 weeks. But the structure of the blood capillary was completely mature (Figure 4).

### 3.3. Immunohistochemistry Assessment

**Figure 5:**
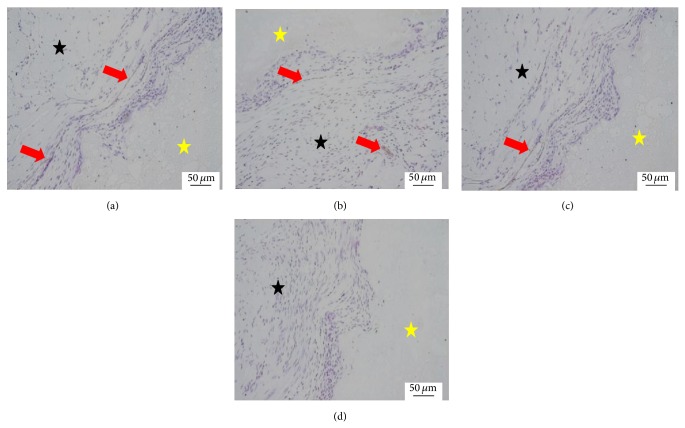
Immunohistochemistry staining of CD31 at 2 weeks (^*^200). (a) The 1 : 1 P/L ratio of CPC-FG; (b) 1 : 0.5 P/L ratio of CPC-FG; (c) 1 : 2 P/L ratio of CPC-FG; (d) pure CPC. The yellow star was represented as the materials and the black star was surrounding muscle tissue. The red arrow was the CD31 staining of new blood vessel.

**Figure 6:**
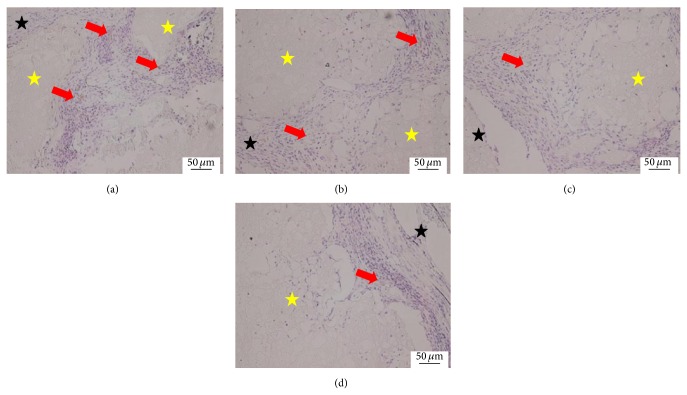
Immunohistochemistry staining of CD31 at 4 weeks (^*^200). (a) The 1 : 1 P/L ratio of CPC-FG; (b) 1 : 0.5 P/L ratio of CPC-FG; (c) 1 : 2 P/L ratio of CPC-FG; (d) pure CPC. The yellow star was represented as the materials and the black star was surrounding muscle tissue. The red arrow was the CD31 staining of new blood vessel.

**Figure 7:**
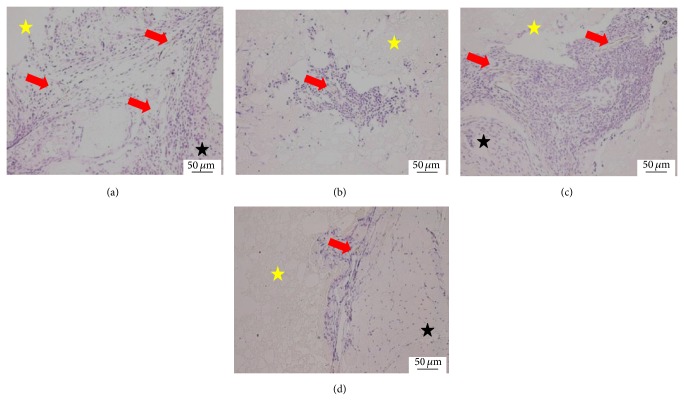
Immunohistochemistry staining of CD31 at 8 weeks (^*^200). (a) The 1 : 1 P/L ratio of CPC-FG; (b) 1 : 0.5 P/L ratio of CPC-FG; (c) 1 : 2 P/L ratio of CPC-FG; (d) pure CPC. The yellow star was represented as the materials and the black star was surrounding muscle tissue. The red arrow was the CD31 staining of new blood vessel.

**Figure 8:**
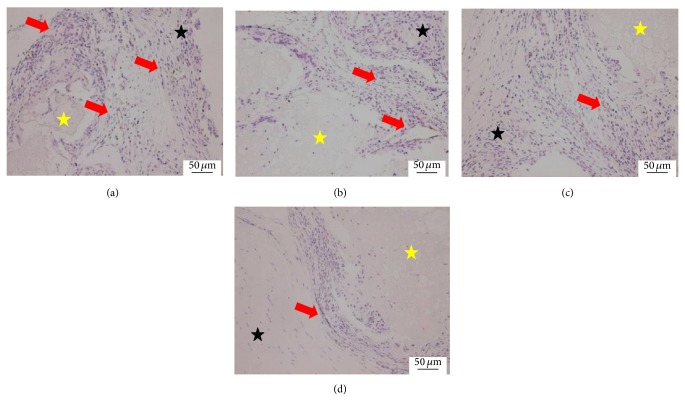
Immunohistochemistry staining of CD31 at 12 weeks (^*^200). (a) The 1 : 1 P/L ratio of CPC-FG; (b) 1 : 0.5 P/L ratio of CPC-FG; (c) 1 : 2 P/L ratio of CPC-FG; (d) pure CPC. The yellow star was represented as the materials and the black star was surrounding muscle tissue. The red arrow was the CD31 staining of new blood vessel.

The positive immunohistochemistry staining for CD31 was found in all groups at week 2 postoperatively with more intensive positive staining in all CPC-FG groups than CPC group (Figure 5). The intensity of positive CD31 staining increased with time and peaked at week 4. In CPC group, the intensity of positive CD31 staining still increased with time (Figure 6). At weeks 8 and 12, the intensity of positive CD31 staining remained steady in all CPC-FG groups and continued to increase with time in CPC group. However, the positive staining of CD31 in CPC group remained less intensive than those of CPC-FG groups at different time points (Figures 7 and 8).

### 3.4. Quantification of Vascular Regeneration

**Figure 9:**
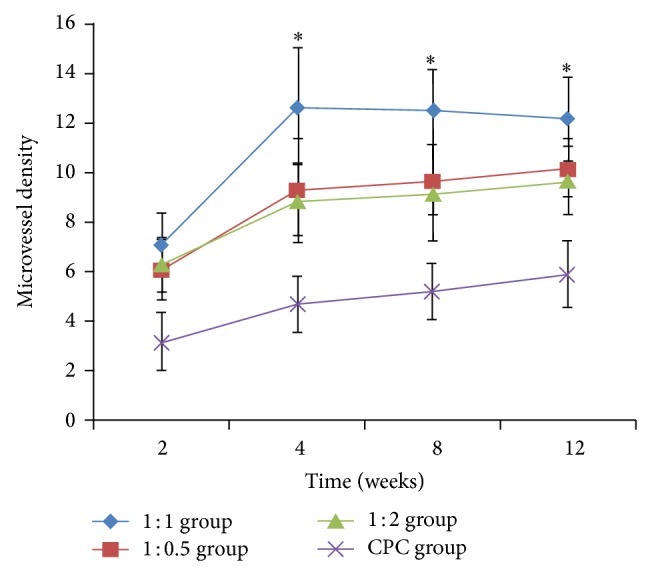
The number of vascular sections in all Groups at 2, 4, 8, and 12 weeks postoperatively. ^*^ *P* < 0.05 versus other groups.

The amounts of blood capillary in CPC group were increased with the time. In three CPC-FG groups, the amounts of blood capillary were rapidly increased to the peak at 4 weeks and then did not significantly change at 8 and 12 weeks (Figure 9). The amounts of blood capillary in three CPC-FG groups were all significantly higher than in CPC groups at 2, 4, 8, and 12 weeks (*P* < 0.05). At 2 weeks, there was no significant differences among three CPC-FG groups (*P* > 0.05). At 4, 8, and 12 weeks, the amounts of blood capillary in 1 : 1 group were significantly higher than in the other two groups (*P* < 0.05), but there were no significantly differences between the 1 : 2 group and the 2 : 1 group (*P* > 0.05).

## 4. Discussion

Angiogenesis is essential for delivery of oxygen, nutrients, soluble factors, and numerous cell types required for reparative processes of bone defect, and the process of vascularization is very important for the osteogenesis and degradation of the bone scaffolds [3–6]. Therefore, an ideal bone scaffolds should support angiogenesis and neovascularization to generate a capillary network with the capacity [7–9]. The CD31 antigen was widely distributed in the surface of blood endothelial cell and can be used to detect the process of angiogenesis [25].

Fibrin glues are composed of fibrinogen and thrombin and can be used in the bone tissue regeneration with their osteoinductive and angiogenic properties [26, 27]. Some studies have proven that the fibrin glues could promote the formation of new capillary network [28]. And the degradation product of fibrin glue can promote the migration of endothelial cell in a suitable concentration [29]. The degradation time of the fibrin glue was about 10 to 14 days in vivo according to previous report [30]. And in our study, the new blood vessel was shown in all CPC-FG groups at 2 weeks and this result was in accordance with the degradation time of fibrin glue. At 4 weeks, the amounts of new blood vessels were increased to the peak and the result was also the same as we expected. Because the fibrin glue was degraded completely and the pore space was formed in the scaffold after 4 weeks, the promotion effect of the fibrin glue was decreased and disappeared finally. So the amounts of new blood vessels remained steady at 8 and 12 weeks and the structure of the blood vessel became more mature with time. But in the CPC scaffolds without fibrin glue, the amount of new blood vessels was increased with time and the process of neovascularization in the scaffold was very slower compared with other CPC-FG groups.

In our study, we found that the ratios of CPC and FG in the scaffold influenced the process of the neovascularization in vivo. At 2 weeks, the amounts of the new blood vessels among all CPC-FG groups were not significantly different, but all of them were higher than the CPC group. At 4, 8, and 12 weeks, the amounts of the new blood vessels in the 1 : 1 group were significantly higher than other groups. According to the previous report, the degradation product of fibrin glue can promote the migration of endothelial cell in a suitable concentration [29]. So the process of the neovascularization was influenced by the ratio of FG in the scaffold. It was not meant that more FG was better for the formation of new blood vessels. In our study, the amounts of the new blood vessels in the 1 : 2 group were lower than in the 1 : 1 group and familiar with the 1 : 0.5 group. But the concrete mechanism of the neovascularization promoted by FG remains uncertain.

In this study, we observed different growth of capillary vessels in different scaffolds. The amounts of new blood vessel peaked at 4 weeks and remained steady at 8 and 12 weeks in all CPC-FG groups. We found that the process of neovascularization in the scaffold with FG was better than in the scaffold without FG at different time points. Therefore, the FG had the angiogenic properties which were in agreement with other studies [26, 27]. Different ratios of FG in the scaffold resulted in different amounts of new blood vessel and CPC-FG composite scaffold at the P/L ratio of 1 : 1 stimulated revascularization better than any other group. This result was in agreement with our previous study about the fact that CPC-FG composite scaffold at the P/L ratio of 1 : 1 stimulated bone regeneration better than any other group [24]. Our study may explain the reason of the fact that P/L ratio of 1 : 1 had a better bone regeneration than other ratios.

## 5. Conclusions

This study demonstrated that the self-setting calcium phosphate cement scaffolds consisting of different proportions of fibrin glue had different angiogenic abilities in vivo. The CPC-FG composite scaffold at the P/L ratio of 1 : 1 had a better angiopoiesis than any other scaffolds and has significant potential as the bioactive material for the treatment of bone defects.
